# Expression Patterns and Potential Biological Roles of Dip2a

**DOI:** 10.1371/journal.pone.0143284

**Published:** 2015-11-25

**Authors:** Luqing Zhang, Humphrey A. Mabwi, Norberto J. Palange, Ruirui Jia, Jun Ma, Fatoumata Binta Bah, Rajiv Kumar Sah, Dan Li, Daji Wang, Fatoumata Binta Maci Bah, Jacques Togo, Honghong Jin, Luying Ban, Xuechao Feng, Yaowu Zheng

**Affiliations:** 1 Transgenic Research Center, School of Life Sciences, Northeast Normal University, Changchun, China; 2 Key Laboratory of Molecular Epigenetics of Ministry of Education, Northeast Normal University, Changchun, China; University of Texas at Austin Dell Medical School, UNITED STATES

## Abstract

Disconnected (disco)-interacting protein 2 homolog A is a member of the DIP2 protein family encoded by *Dip2a* gene. *Dip2a* expression pattern has never been systematically studied. Functions of *Dip2a* in embryonic development and adult are not known. To investigate *Dip2a* gene expression and function in embryo and adult, a *Dip2a-LacZ* mouse model was generated by insertion of *β-Gal* cDNA after *Dip2a* promoter using CRISPR/Cas9 technology. *Dip2a-LacZ* mouse was designed to be a *lacZ* reporter mouse as well as a *Dip2a* knockout mouse. Heterozygous mice were used to study endogenous *Dip2a* expression and homozygotes to study DIP2A-associated structure and function. LacZ staining indicated that *Dip2a* is broadly expressed in neuronal, reproductive and vascular tissues, as well as in heart, kidney, liver and lung. Results demonstrate that *Dip2a* is expressed in ectoderm-derived tissues in developing embryos. Adult tissues showed rich staining in neurons, mesenchymal, endothelial, smooth muscle cells and cardiomyocytes by cell types. The expression pattern highly overlaps with FSTL1 and supports previous report that DIP2A to be potential receptor of FSTL1 and its protective roles of cardiomyocytes. Broad and intense embryonic and adult expression of *Dip2a* has implied their multiple structural and physiological roles.

## Introduction

DIP2A is a member of Disconnected (disco)-interacting protein 2 (DIP2) family with other two isoforms, DIP2B and DIP2C. Bioinformatic analysis using Predict Protein and Homolo Gene suggested that DIP2A is a type I receptor molecule with DMAP, CaiC and AMP-binding domains [1]. Mukhopadhyay reported that DIP2 homologs are evolutionarily conserved in organisms from *C*. *elegans* to humans. DIP2A proteins may exert their signaling roles as receptors in prokaryotes and eukaryotes and may provide positional cues for axon path finding and patterning [2]. Although expression pattern and physiological function of DIP2A are currently unknown, previous studies have indicated that *Dip2a* expression is restricted to brain in mouse embryos, including neocortex, striatum and thalamus using Northern and *in situ* hybridization.

LacZ enzyme activity can be easily visualized by X-Gal substrate staining [3]. Transgenic expression of β-Galactosidase gene (*LacZ*) has been widely used to trace endogenous gene expression and to predict potential biologic functions. In order to systematically investigate *Dip2a* gene expression in embryonic development and in adult tissues, we generated *Dip2a-LacZ* mouse using CRISPR/Cas9 system [4]. Embryos from E9.5, E11.5, E12.5, E15.5 and adult tissues from *Dip2a* ^*LacZ/+*^ and *Dip2a* ^*LacZ/LacZ*^ mouse were harvested. To identify specific *Dip2a* expression, whole-mount and frozen sections were stained with X-gal in parallel with wild type littermate controls.

## Materials and Methods

All the general chemicals were from Sigma, USA and enzymes from Takara, Dalian, China.

### Animals

All animals were maintained in a clean facility in Northeast Normal University. Mice were kept in IVC cages (5 per cage) with free access of food and water, at 20°C and 50 ± 20% relative humidity under a 12:12h light:dark cycles and pathogen free conditions. Mice were anesthetized before sacrificing with 1% pentobarbital at a dose of 10 mg/kg. All procedures were based on Guide for Care and Use of Laboratory Animals of National Institutes of Health and approved by Institutional Animal Care and Use Committee of Northeast Normal University (NENU/IACUC, AP2013011). C57BL/6J mice were purchased from Vital River (Beijing, China). *Dip2a-LacZ* mice were generated using CRISPR/Cas9 technology as described previously [4]. *B6J*.*129S4/SvJaeSor-Gt(ROSA)26Sor* ^*tm1(FLP1)Dym*^ */JNju* were obtained from Nanjing Biomedical Research Institute of Nanjing University, China.

### Genotyping

Embryos, pups and adult mice were genotyped for *LacZ* insertion by PCR. Genomic DNA was extracted from yolk sacks, embryonic limbs and tail tips. Samples were digested with GNT-K buffer at 55°C overnight [5]. Tail lysates were diluted and boiled for 15min. PCR was performed at 94°C for 2min followed by 30 cycles of denaturation at 94°C for 30sec, annealing at 60°C for 30sec and extension at 72°C for 30sec. Final extension was at 72°C for 5min and hold at 4°C. PCR product of 250bp was identified on 0.8% agarose gel. The primer sequences used for *LacZ* allele were ZF5'-ACCACACCTCCTGCTGTATAC-3' and ZR5'-ACGACGGGATCATCGCGAGCCAT-3'. Primers WF5'-GGGTCACCTGGGCGACATTGA-3' and WR5'-TCACCTTCG-GACAGCTCCAGCT-3' were used for wild type allele using GC-rich buffer I (Takara biotechnology) and slow down PCR program [6]. *Neo* gene was PCR amplified using primers NF5'-AGCTGGGGCTCGACTAGAGCTT-3', NR5'-TCACCTTCGGACA-GCTCCAGCT-3' and *Flipase* gene using primers FF5'-AAAGCATCTGGGAGAT-CACTGAG-3' and FR5'-TATACAAGTGGATCGATCCTAC-3' respectively.

### Whole mount embryos and adult tissues collection and LacZ staining

Embryos and adult tissues were dissected from of *Dip2a* ^*LacZ/+*^ mice. Adult mice were anesthetized with 1% pentobarbital at a dose of 10mg/kg, whereas pups of 3 week old and pregnant dams were decapitated. Embryos were dissected from pregnant dams in ice-cold PBS. Whole mount staining of E9.5, E11.5, E12.5, E15.5 and adult tissues were fixed in 2% PFA, 0.25% glutaraldehyde and 0.01% NP40 in PBS with agitation at 4°C for 30min for embryos and 1–2h for adult tissues. Samples were then washed 3 times, 15min each in large volume of rinse buffer (2mM MgCl_2_, 0.02% NP40 and 0.01% Na-deoxycholate in PBS) and stained in 30mM K_3_Fe(CN)_6_, 30mM K_4_Fe(CN)_6_3H_2_O, 2mM MgCl_2_, 0.01% Na-deoxycholate, 0.02% NP40 and 1mg/ml 5-bromo-4-chloro-3-indolyl-β-D-galactopyranoside (X-Gal) in PBS at 37°C for 6–12hrs. Embryos were washed 3 times in large volume of PBS for 20min each, post-fixed overnight in 4% PFA with agitation at 4°C, followed by 3 times wash with PBS. Stained tissues and embryos were stored in 70% glycerol at 4°C and photographed with Olympus microscope (SZX-ILLB2-200, Japan) and Canon digital camera (DSI26431, Japan).

### Frozen section and LacZ staining

Adult mice were anesthetized with 1% pentobarbital at a dose of 10mg/kg. Tissues were collected in ice cold PBS, fixed in 2% PFA, 0.25% glutaraldehyde and 0.01% NP40 in PBS for 1–2h with agitation at 4°C. Samples were washed 3 times, 15min each in PBS and in 20% sucrose overnight at 4°C except eyes and embryos were incubated longer. Tissues were embedded in OCT compound (Tissue-Tek, USA) and frozen at -80°C. Serial cryosections were cut at 25μm thickness and collected onto Superfrost^TM^ Plus microscope slides. After drying on a slide warmer for 2h, sections were fixed with 2% PFA in PBS for 10min at room temperature, washed in 2mM MgCl_2_, 0.02% NP40, 0.01% Na-deoxycholate and were fully immersed in LacZ staining buffer (30mM K_3_Fe(CN)_6_, 30mM K_4_Fe(CN)3H_2_O, 2mM MgCl_2_, 0.02% NP40, 0.01% Na-deoxycholate and 1mg/ml X-Gal in PBS) at 37°C overnight. After staining, sections were fixed in 4% PFA for 5min and washed in PBS. Sections were counter stained in 0.25% eosin solution. Images were taken using Olympus microscope (SXL-ILLB2-200, Japan) and Canon digital camera (DSI26431, Japan).

### RNA isolation and real time PCR

Total RNA was isolated using Trizol reagent (Invitrogen, USA) according to manufacturer’s instruction. One microgram total RNA was reverse transcribed using Primescript^TM^II cDNA Synthesis Kit (Takara biotechnology, Dalian, China). Quantitative real time PCR was performed using Light Cycler 480 sequence detection system (Roche, Indianapolis, USA) and SYBR II premix (Takara). All results were normalized to 18S ribosomal RNA and relative quantification was calculated using comparative threshold cycle (ΔΔ^Ct^) values for each biological replicate. Primers mDip2A-QPCRF: ACAGGAGCATTGCAGAGTGTG and mDip2A-QPCRR: TGGTTCCTACAGCCAG-CTCTGTC were used.

### Immunofluorescence and Immunohistochemistry

Tissues were rinsed in PBS and fixed in buffer as described previously. Samples were transferred to 20% sucrose in PBS at 4°C overnight prior to embedding in OCT. Tissues were quick frozen and stored at -80°C. Brain was cut into 40um slices, washed in PBS for 5min and antigen retrieved in Na-citrate (pH6.0) at 96°C for 5min. Slices were cooled to room temperature and washed 3 times in PBS, 5min each. Brain slices were carefully attached onto slides. Sections were further incubated with primary antibodies: mouse anti-GFAP, 1:1000 dilution (Sigma, G3893); mouse anti-NeuN 1:1000 dilution (Millipore, MAB377); chicken polyclonal anti-β-galactosidase, 1:1000 dilution (Abcam, ab9361) in a humidified box at 4°C overnight. Primary antibodies were diluted in PBS containing 0.2% Triton X-100 and 2% goat serum and 200μl was applied to each slide. Slides were moved to room temperature for 30min followed by 6 times, 5min each PBST washes. Fluorescent secondary antibody incubation was carried out at room temperature for 1h (Alexa Fluor 594 donkey anti-mouse, A11040, Invitrogen, USA; Alexa Fluor 488 goat anti-chicken, A11008, Invitrogen, USA). All secondary antibodies were diluted in PBS at 1:200. Sections were washed in PBST 6 times, 5min each and followed by DAPI (4,6-diamidino-2-phenylindole) staining (1mg/ml) and PBST rinse. Fluorescent images were collected using fluorescent microscope (Nikon, Japan).

## Results

### Generation of *Dip2a-LacZ* reporter mouse

*LacZ* reporter mice have made huge contribution in revealing gene expression patterns in developing embryos and in adults. *Dip2a* is one of the genes that have never been systematically studied neither on expression nor functions although many important biological roles have been suggested. Using CRISPR/Cas9 system, we have generated nuclear *LacZ* knock-in mice by co-injection of *LacZ-FRT-PGK-neomycin-FRT* donor plasmid and *Cas9/sgRNA* plasmid [4]. After germline transmission, *Dip2a-LacZ-neo* mouse was crossed with transgenic mice that express Flipase to excise *Neo* cassette, resulting to *Dip2a* ^*LacZ/+*^ mice [7]. Genetic background of *Dip2a* ^*LacZ/+*^ mice was switched to C57BL/6J by continued crossing heterozygous *Dip2a* ^*LacZ/+*^ mice with C57BL/6J mice.

### *Dip2a-LacZ* expression in developing embryos

**Fig 1:**
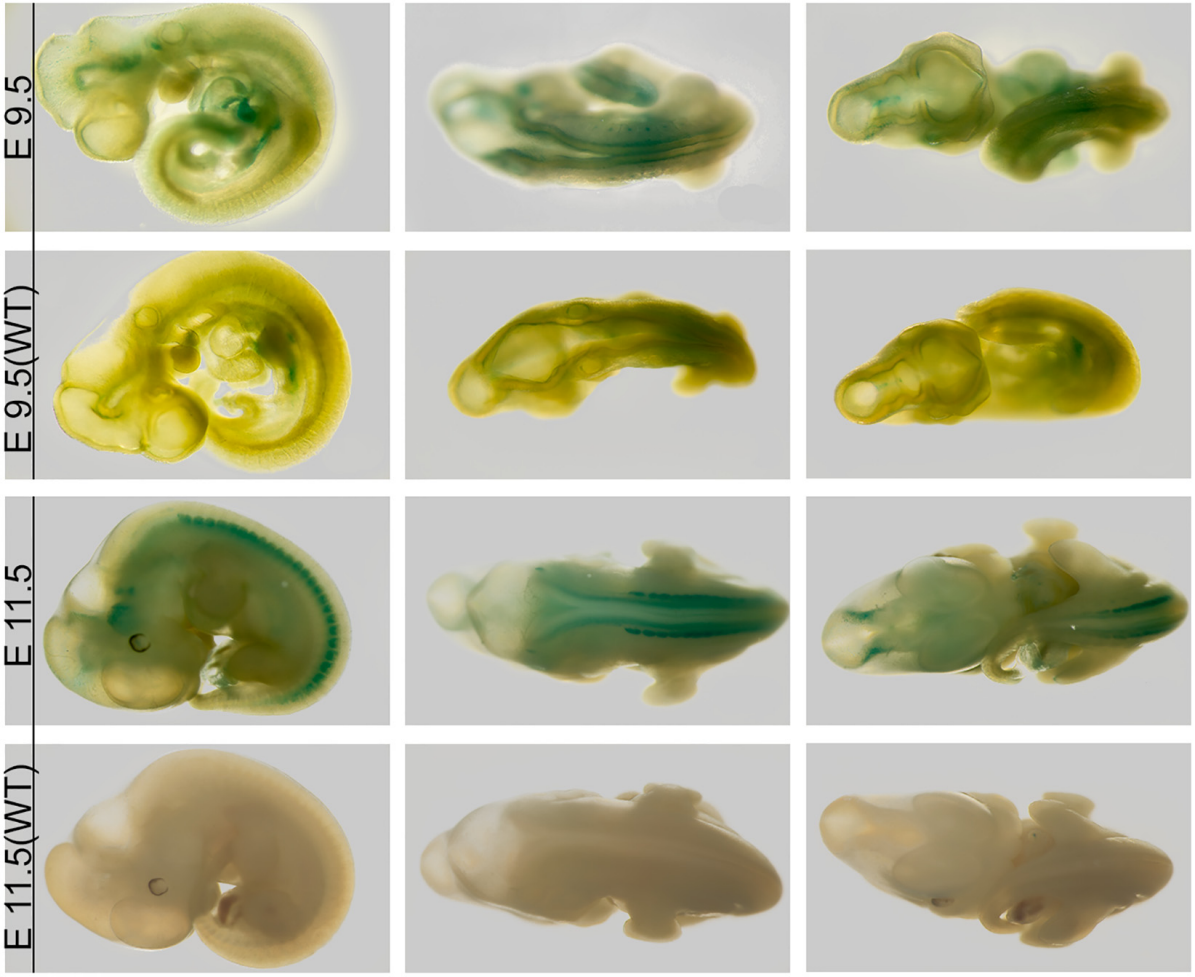
E9.5 and E11.5 LacZ staining. Control wild type embryos are labeled (WT).

**Fig 2:**
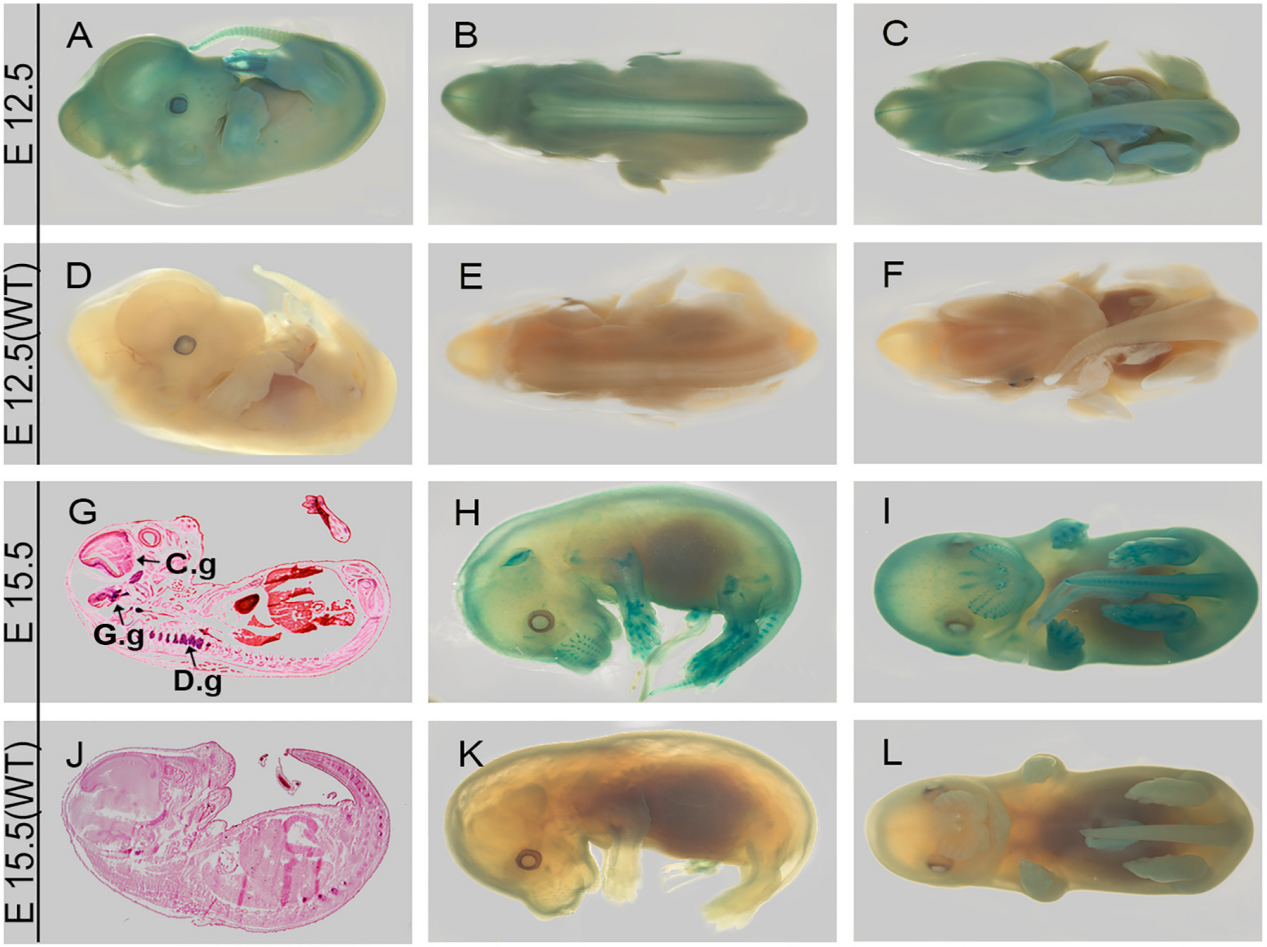
E12.5 and E15.5 LacZ staining. Coronal section of E15.5 (G) showed signals in cranial ganglion (C.g), gasserian ganglion (G.g) and dorsal root ganglion (D.g).

We first investigated LacZ expression in developing embryos at E9.5, E11.5, E12.5 and E15.5. Whole mount staining of E9.5 and E11.5 embryos revealed strong signals in developing spinal nervous system (Fig 1). Snout merkel cells at base of whiskers, both forelimbs and hind limbs are also LacZ positive at E12.5 and E15.5 (Fig 2A–2C, 2H and 2I). Phalanges of wrists and toes show stronger signal in anterior region than posterior regions (Fig 2H and 2I). Staining of 25μm coronal sections from E15.5 showed developing nervous system including dorsal root ganglion (D.g), cranial ganglion (C.g) and gasserian ganglion (G.g) were LacZ positive (Fig 2G). Results demonstrate that *Dip2a* is expressed in ectoderm-derived tissues in developing embryos.

### *Dip2a-LacZ* expression in Nervous system

*Dip2a-LacZ* staining in different adult tissues revealed strong and broad signals in nervous, reproductive systems. Less broad signals were identified in vascular, respiratory, urinary, endocrine and also in digestive systems.

#### LacZ expression in brain

**Fig 3:**
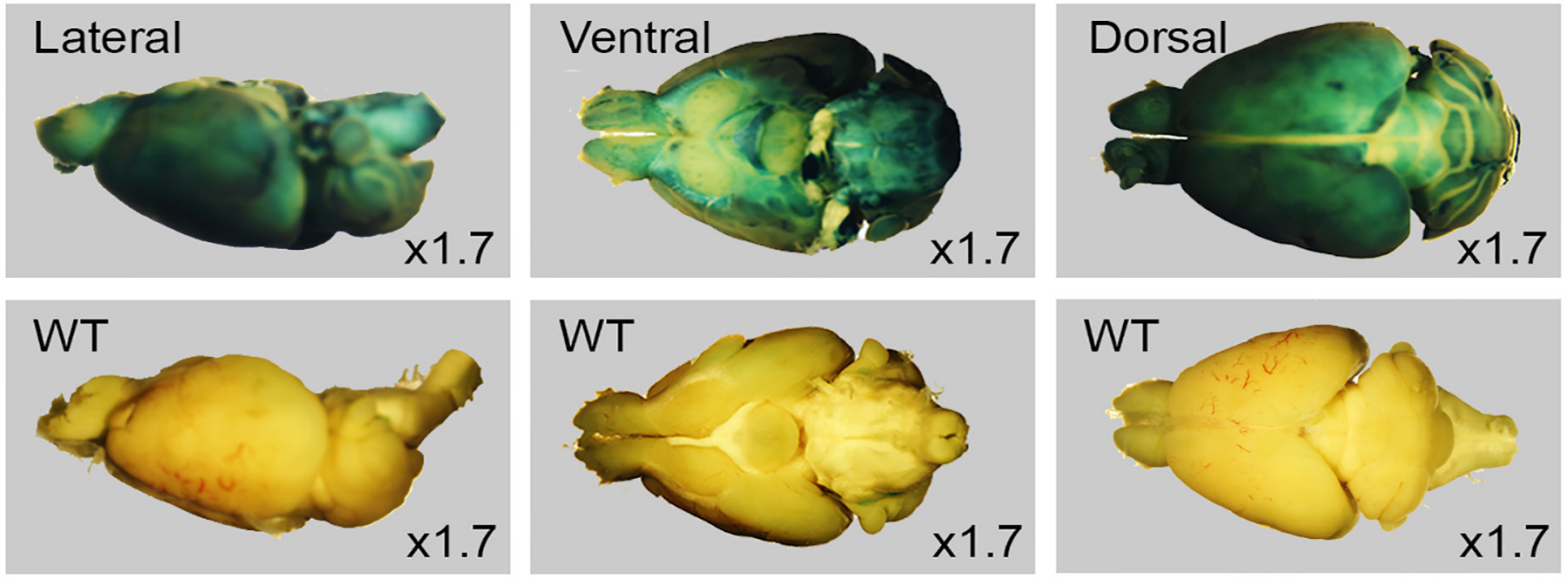
Whole mount LacZ staining of 8 week old adult brain. Control wild type labeled WT.

**Fig 4:**
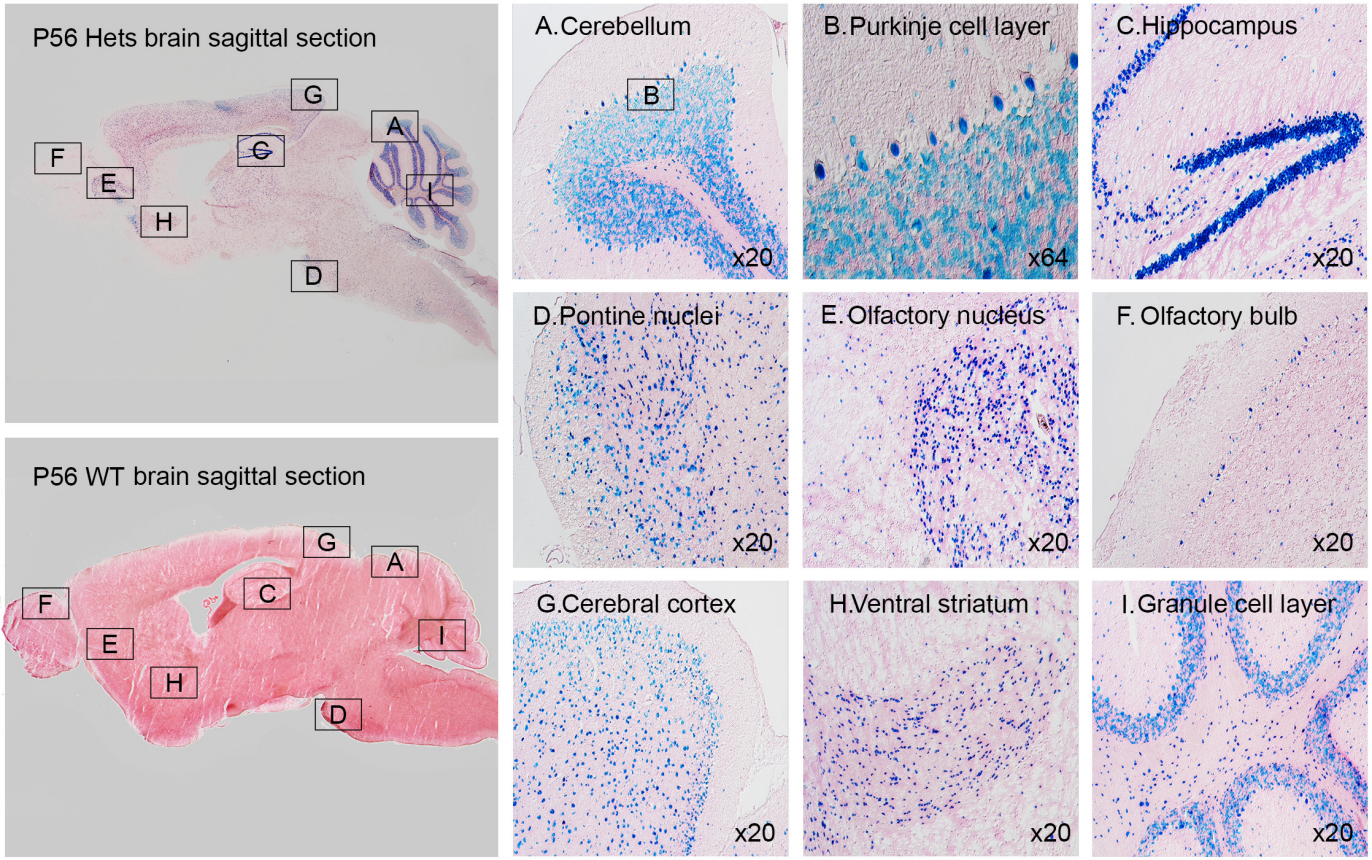
Sagittal sections of LacZ stained 8 week old adult brain.

**Fig 5:**
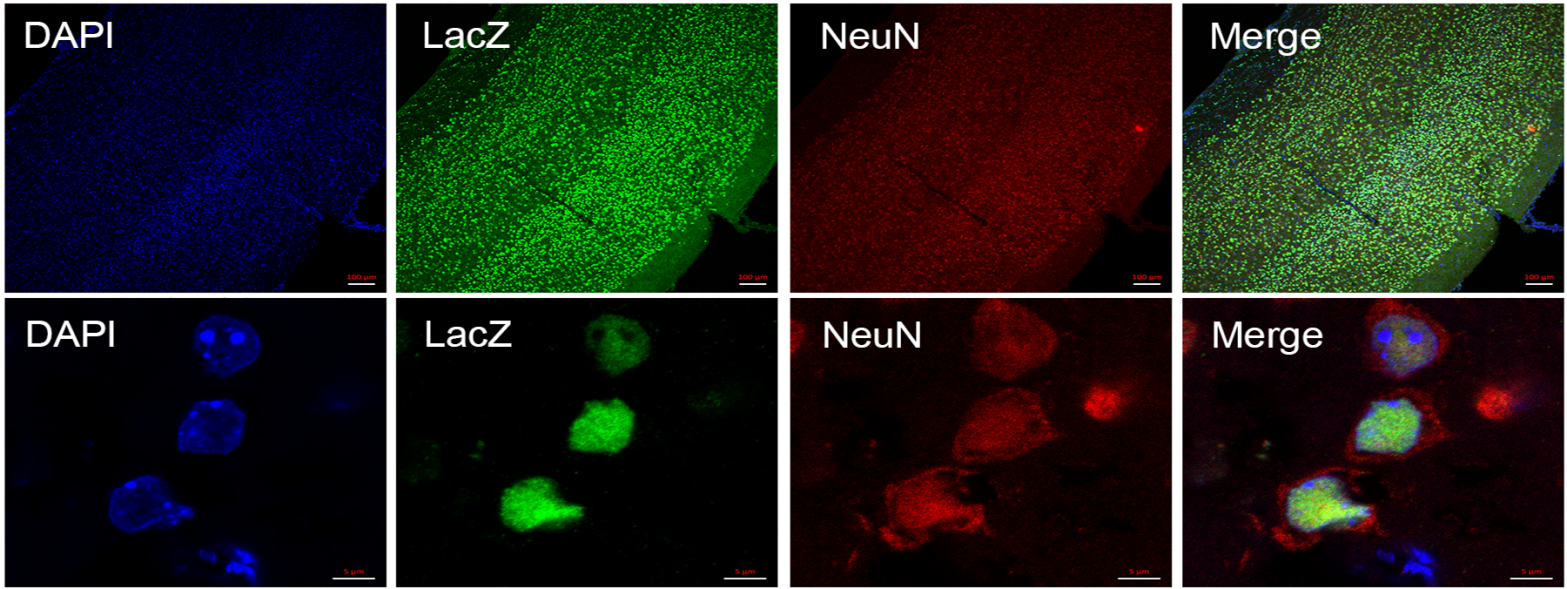
Immunofluorescent staining of 8 week old adult brain section. Cortex region of brain stained using anti-NeuN (Red), a marker specific to neuron, anti-LacZ (Green) and nucleus stained with DAPI (Blue). Top and bottom rows are low and high magnification respectively.

**Fig 6:**
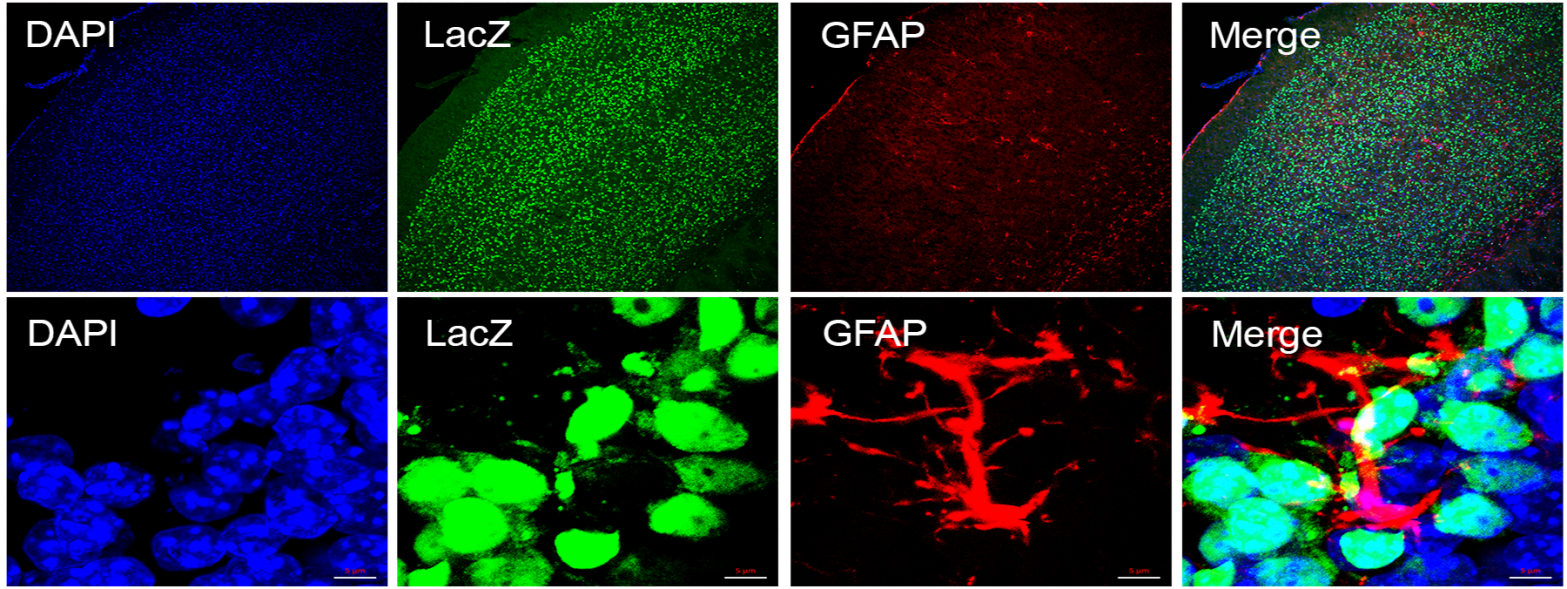
Immunofluorescent staining of adult brain section. Glial cells were stained with anti-GFAP (red), anti-LacZ stained green and nucleus stained with DAPI (Blue). Top and bottom rows are low and high magnification respectively.

Whole mount staining of ventral, lateral and dorsal brain orientation demonstrated strong signals in brain (Fig 3). No staining was observed in control littermates. Staining of 25μm sagittal slices of whole mount brain (Fig 4) showed signals in cerebellum (Fig 4A), purkinje cell layer (Fig 4B), hippocampus (Fig 4C), pontine nuclei (Fig 4D), anterior olfactory nucleus (Fig 4E), olfactory bulb (Fig 4F) and midbrain including cerebral cortex (Fig 4G), striatum (Fig 4H) and granule cell layers (Fig 4I). Immunofluorescent staining using neuron cell type-specific markers NeuN and GFAP revealed that neurons are positive (Fig 5) while glial cells are negative for LacZ expression (Fig 6). Since nuclear lacZ was used to generate *Dip2a-LacZ* mice, perfect merge of LacZ and DAPI were seen.

#### Retina

**Fig 7:**
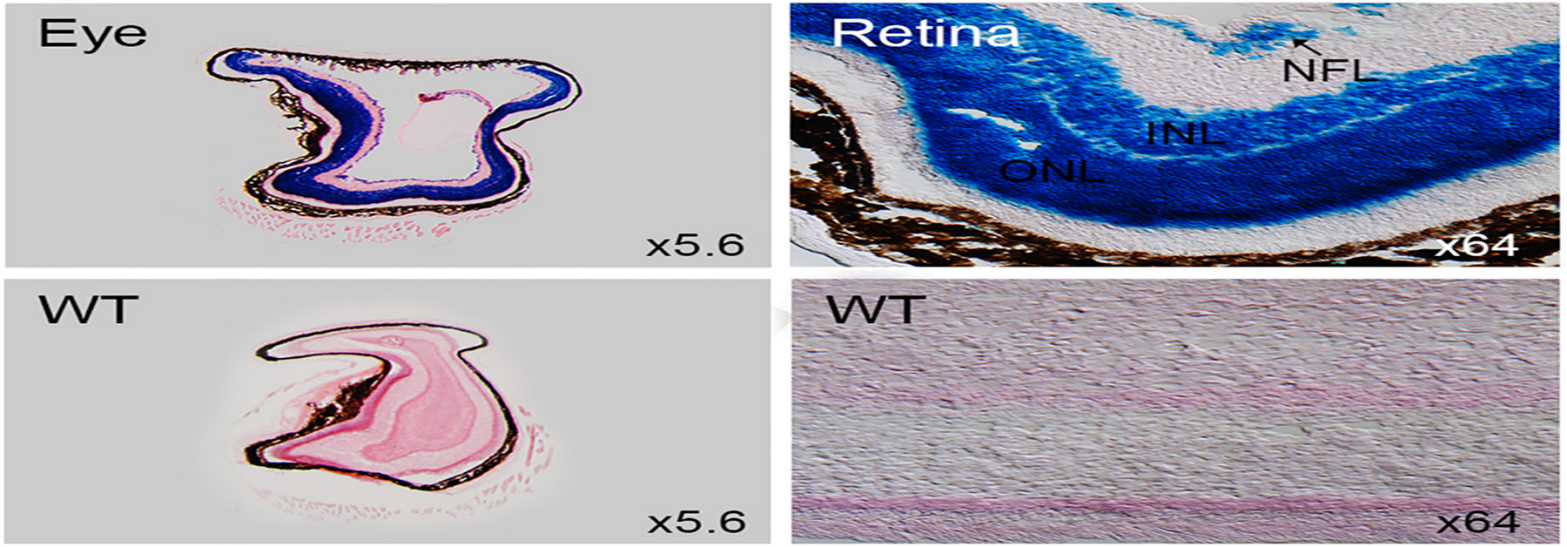
LacZ staining of 8 week old adult retina. Sagittal sections of 8 week old adult eye are stained for LacZ expression. LacZ positive retina (top row) showed strong signals in optic nerve fiber layer (NFL), inner nuclear layer (INL) and outer nuclear layer (ONL).

*Dip2a-LacZ* was strongly expressed in retinal ganglion cells (Fig 7) within optic nerve fiber layer (NFL), inner nuclear layer (INL) and outer nuclear layer (ONL).

#### Spinal cord

**Fig 8:**
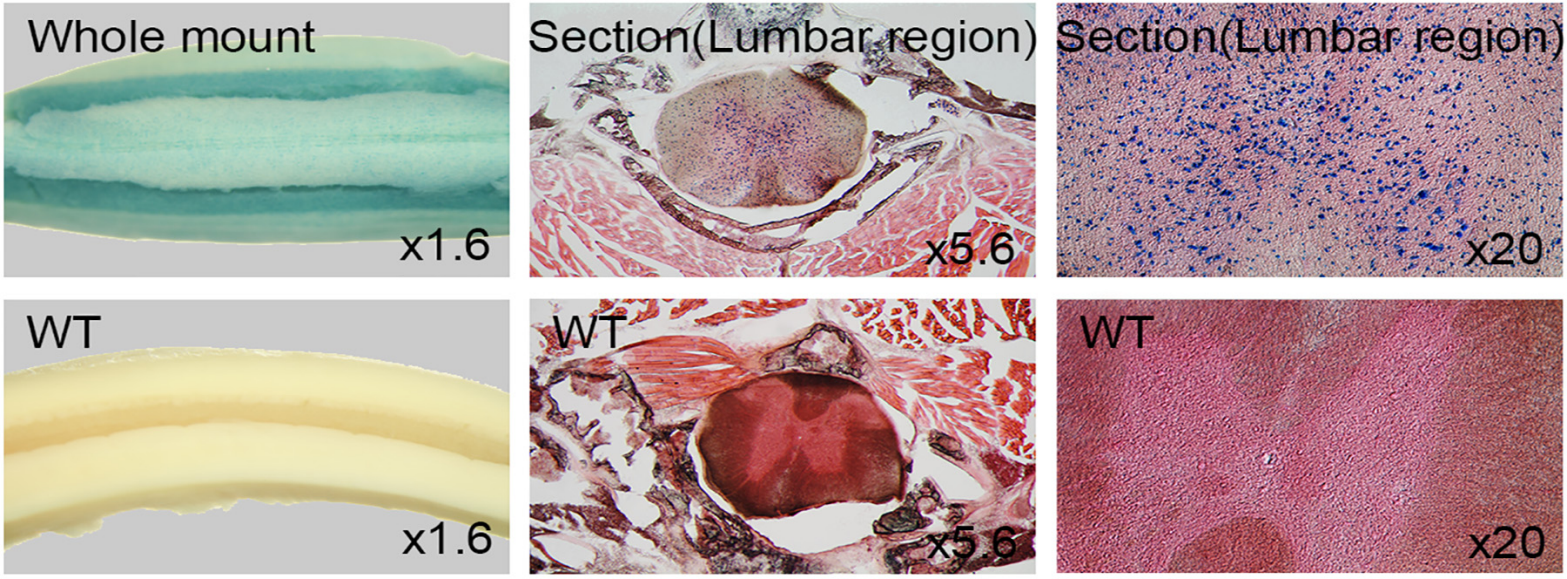
LacZ staining of adult spinal cord. Whole mount staining of 8 week old adult spinal cords (left column), coronal section of lumbar region (middle column) and high magnification of central canal indicate positive LacZ staining.

Spinal cord forms part of central nervous system extending from brain towards posterior end of vertebral column. Whole mount LacZ staining revealed strong signals throughout entire spinal cord. Coronal sections of 25μm thick lumbar region showed that cells surrounding central canal including neurons expressed *Dip2a* (Fig 8). This strong signal in central neuron system is consistent from embryonic development stages to adult.

### Cardiovascular system

**Fig 9:**
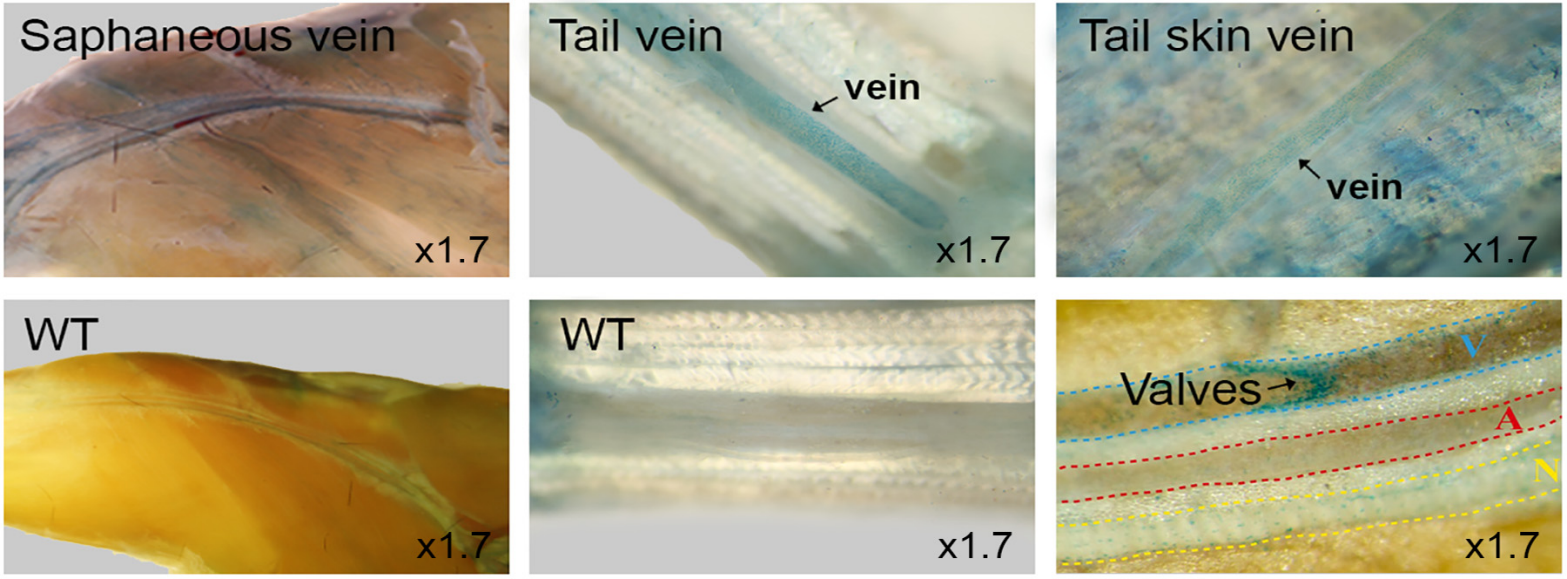
Vein staining of 8 week old adult *Dip2a-LacZ* mice. Saphenous veins, tail veins, tail skin veins and valves were stained positive while control (WT) was negative.

**Fig 10:**
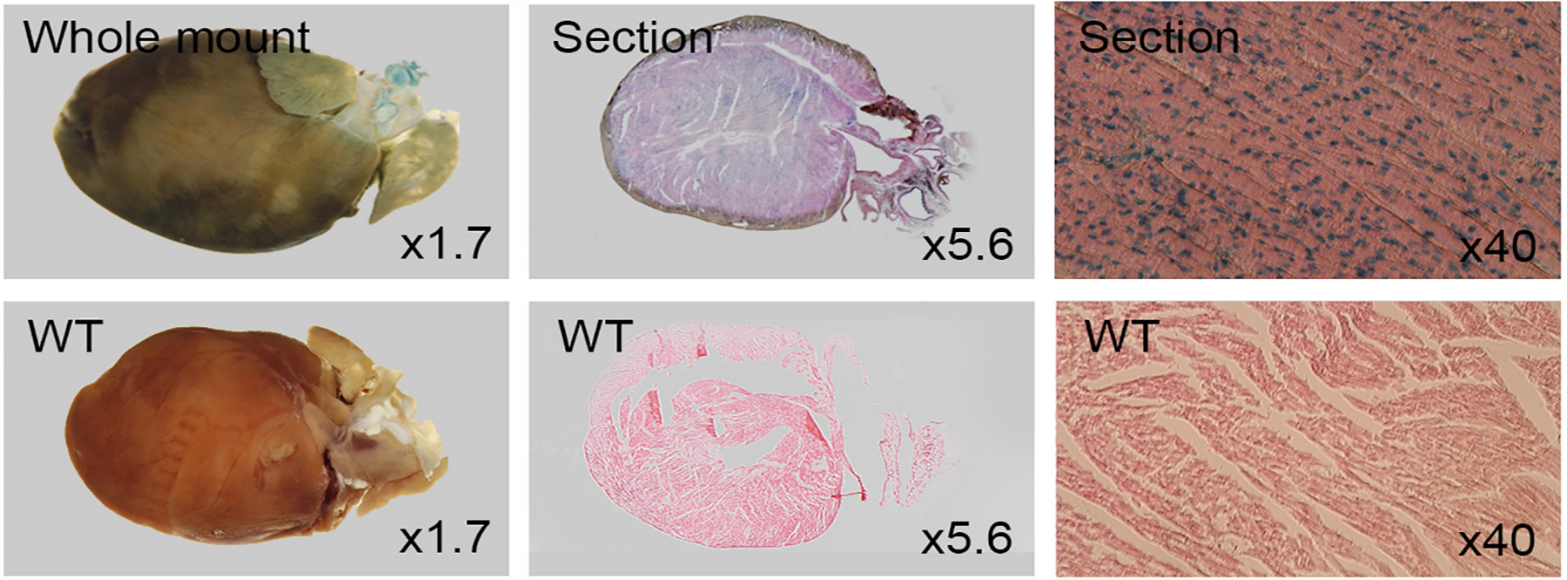
LacZ staining of 8 week old adult heart. Uniform staining in cardiomyocytes while control showed negative. Whole mount (left), section (middle) and high magnification (right).

Dip2a-LacZ is expressed strongly in veins. Whole mount staining of hind limb reveals staining of hind limb saphenous vein, tail vein and tail skin vein (Fig 9). Valves within saphenous vein were positive for LacZ. No staining was observed in wild type control. Strong LacZ activity was observed in cardiomyocytes (Fig 10).

### Reproductive system

Male and female reproductive organs were investigated and LacZ expression was identified in testis, seminal vesicle and ovary.

### Male reproductive tissues

**Fig 11:**
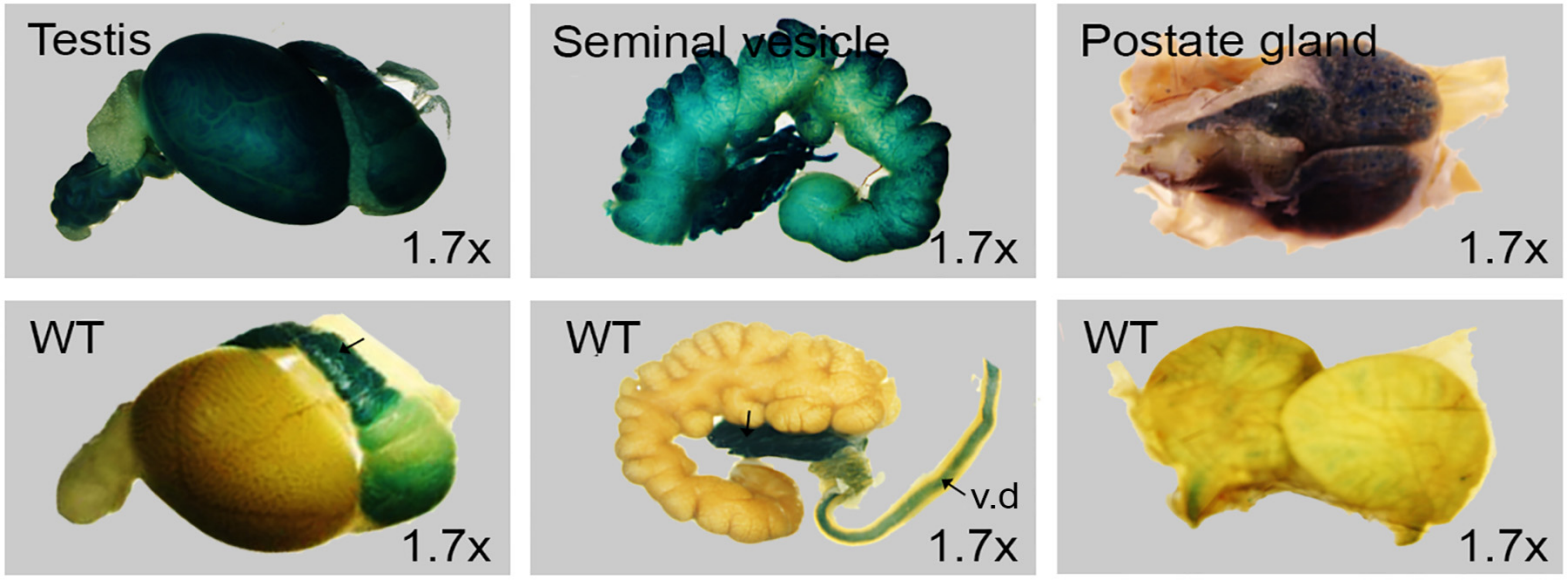
Whole mount LacZ staining of 8 week old male reproductive organs. Testis (left), seminal vesicle (middle) and prostate gland (right) stained in parallel with wild type littermates controls (bottom row). Non-specific staining seen in epididymis tail region (arrow) and vas deferens (v.d).

**Fig 12:**
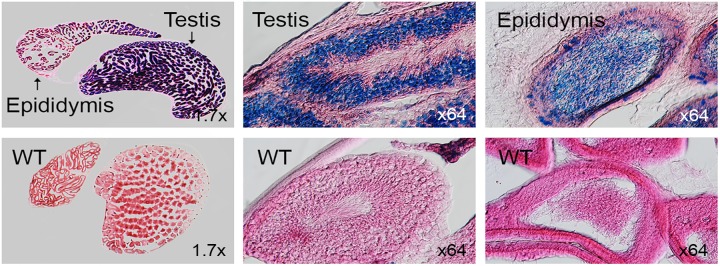
Coronal sections of LacZ-stained 8 week old male testis and epididymis. WT represents controls.

**Fig 13:**
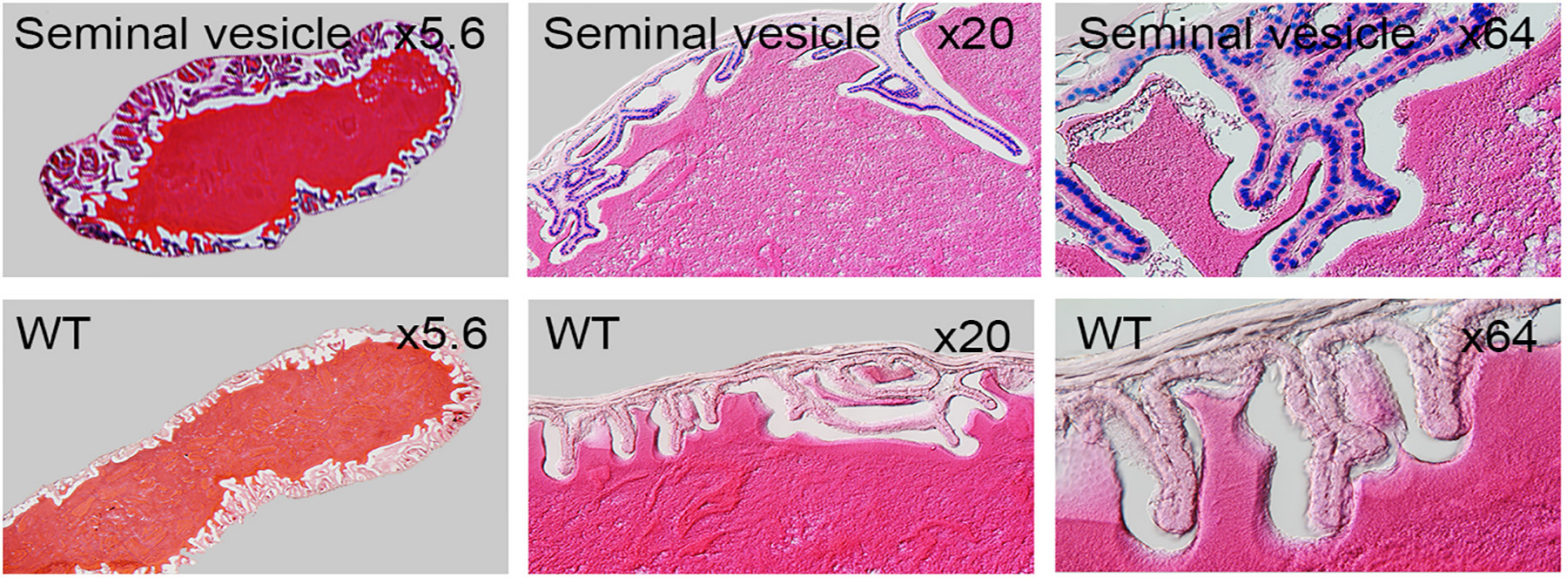
Coronal sections of 8 week old male LacZ-stained seminal vesicle. WT represents controls.

LacZ staining in testis, seminal vesicle and prostate gland revealed strong expression while nonspecific staining was observed in epididymis and vas deferens (Fig 11). Upon sectioning of testis, intense signal was seen in spermatocytes from both testis and epididymis (Fig 12). Strong signal observed in seminiferous epithelium comprising of convoluted seminiferous tubules which houses spermatocytes, spermatids, sustentacular, leyding and sertoli cells. Specific staining was observed in epididymis head region. Secretory granules of seminal vesicle housed in mucosal folds were strongly positive (Fig 13). No staining was detected in interstitial tissue and eonophilic secretion of seminal vesicle. Epididymis and vas deferens to seminal vesicles showed high endogenous β-Galactosidase activities and may mask specific signals in these regions. Testis is negative for LacZ activity before 3 weeks of age (data not shown).

### Female reproductive tissues

**Fig 14:**
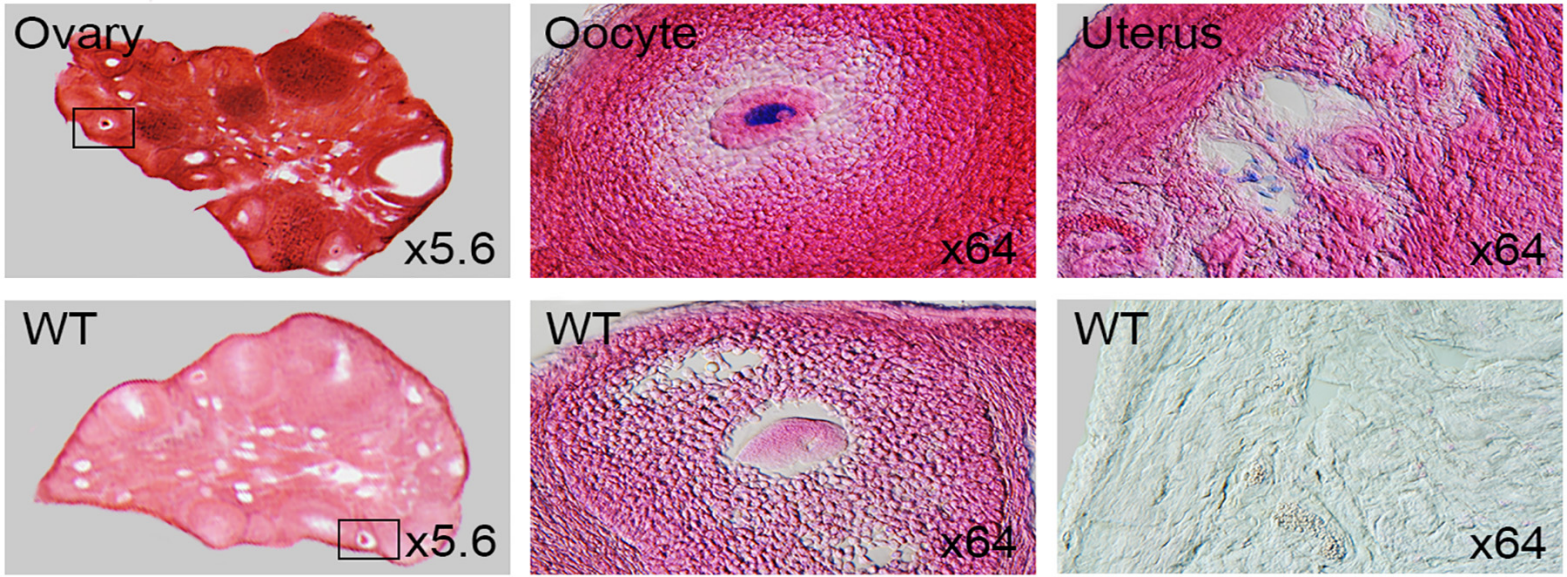
Coronal sections of 8 week old LacZ-stained female reproductive organs. WT represents controls where wild type uterus was not counter stained with Eosin.

*Dip2a-LacZ* was strongly expressed in ovary follicles and oocyte. Weaker signal was found in endometrium of uterus (Fig 14). Comparing to female reproductive organs, *Dip2a-LacZ* expression in male reproductive tissues is much stronger.

### LacZ expression in kidney, lung, tongue, liver and gut

**Fig 15:**
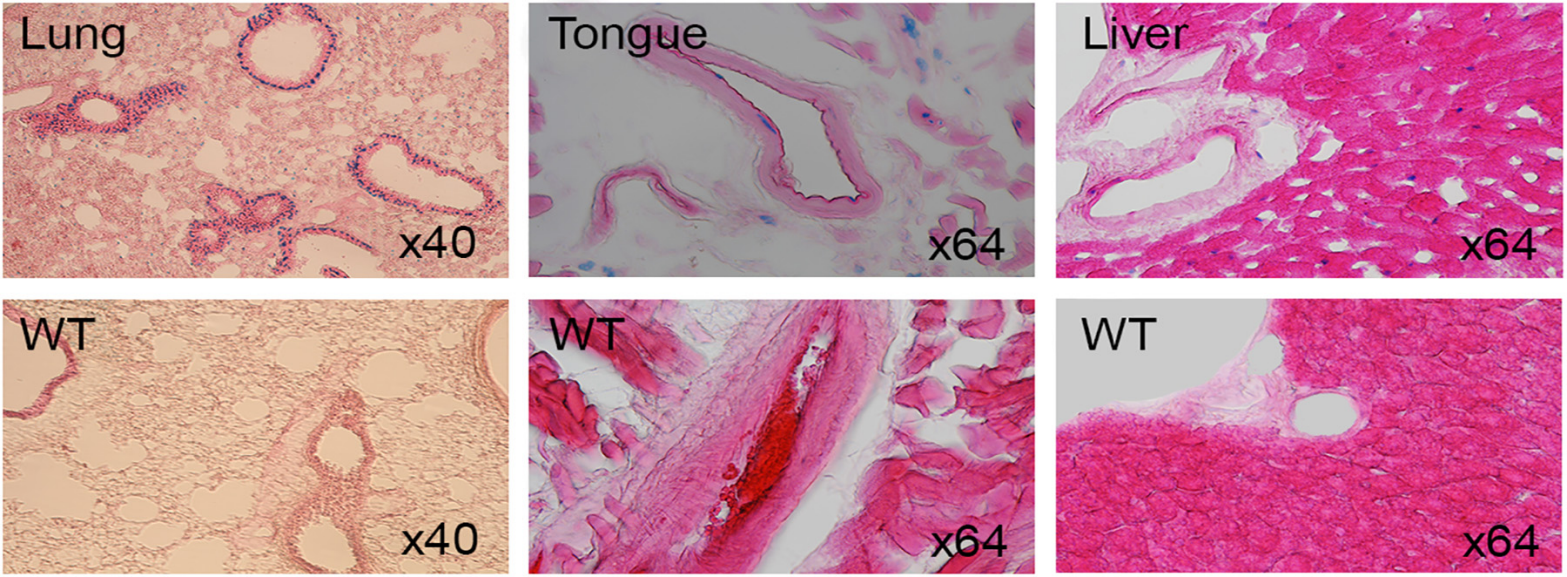
Section of LacZ-stained 8 week old adult lung, tongue and liver. WT represents wild type control.

**Fig 16:**
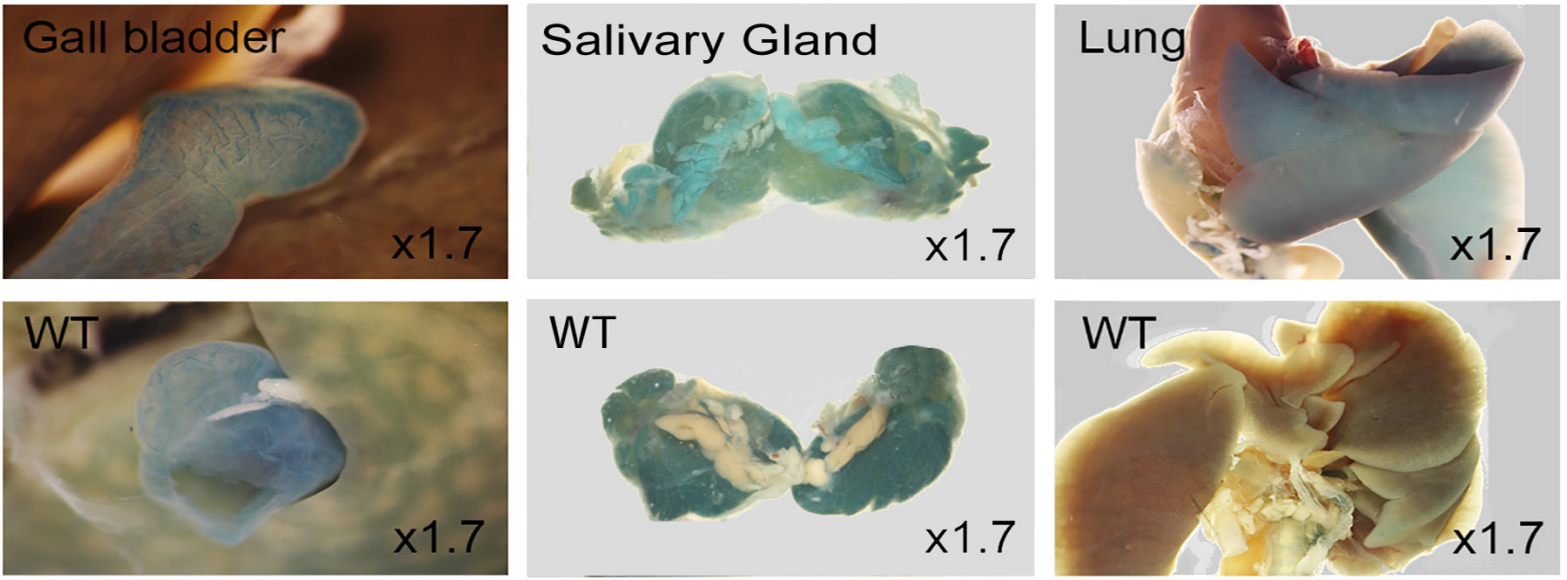
Whole mount staining of 8 week old mice gall bladder, salivary gland and lung. WT represents controls. Endogenous β-Galactosidase activity was observed in the gall bladder and salivary gland.

**Fig 17:**
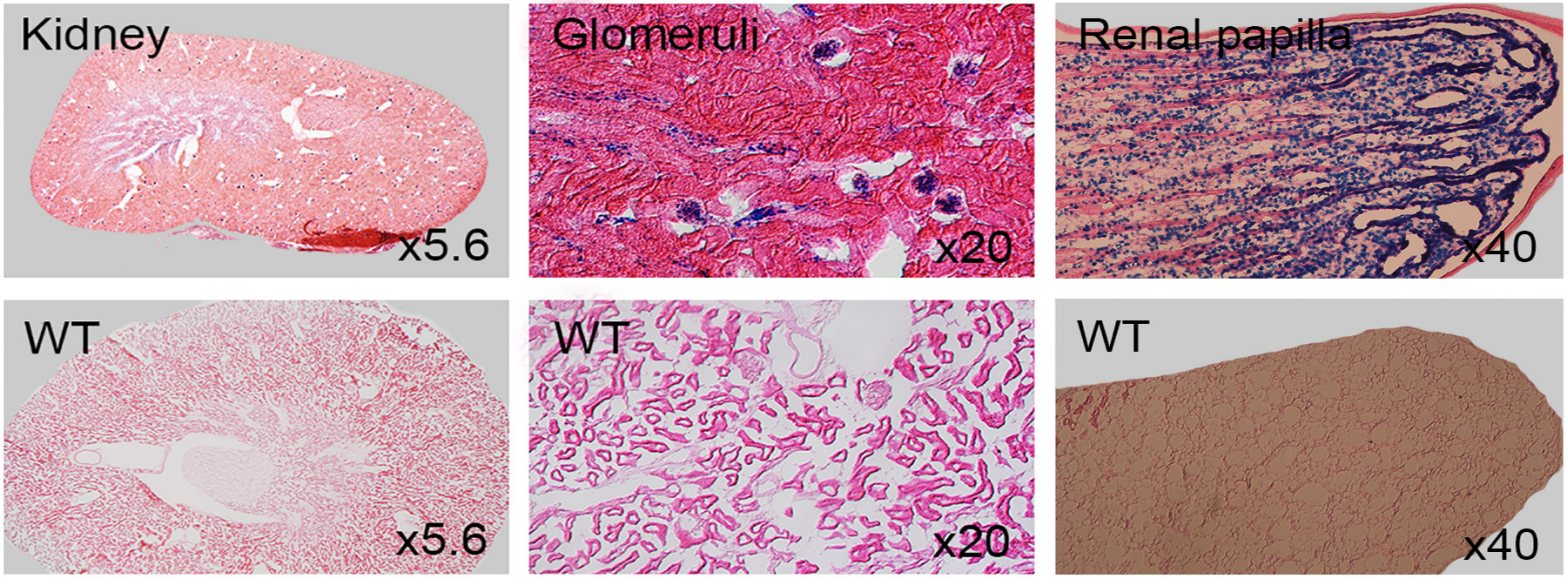
Coronal sections of 8 week old adult LacZ-stained kidney. WT represents controls.

**Fig 18:**
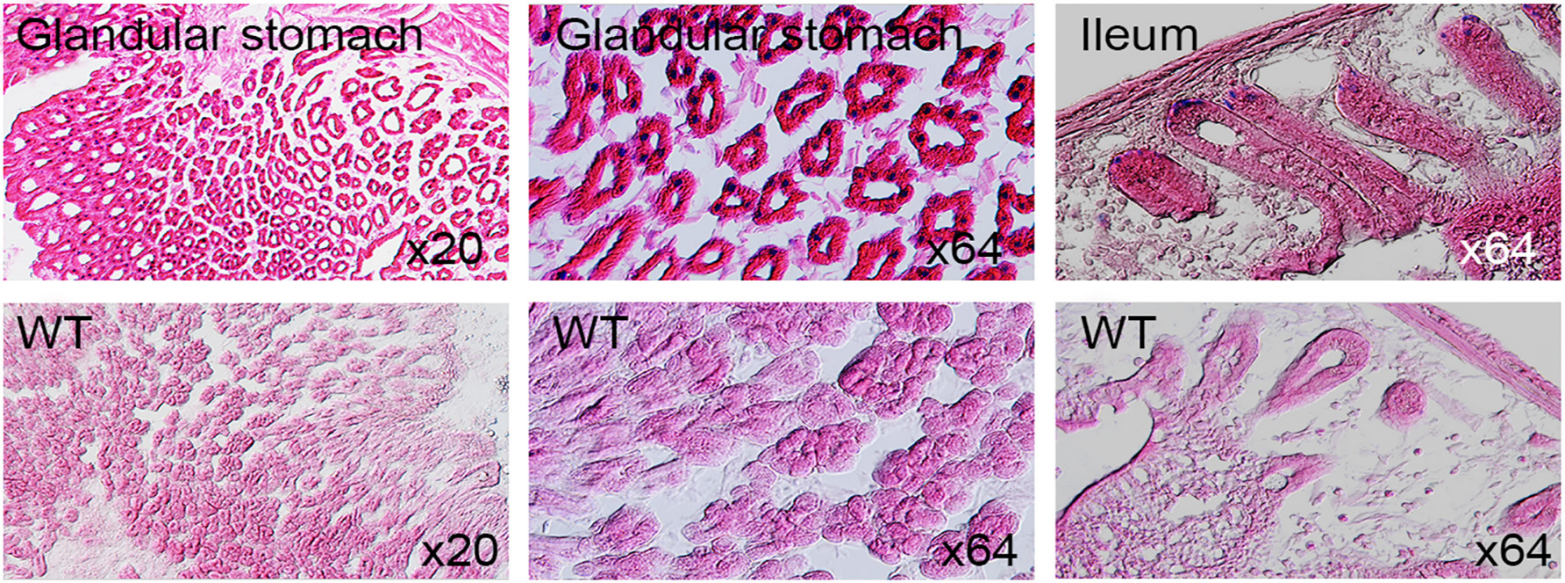
Coronal sections of LacZ-stained 8 week old adult stomach and gut. WT represents controls.

Postnatal tissues from 3 to 8 weeks of age were stained for LacZ expression. Signals were found in liver, lung and tongue (Fig 15). Both specific and non-specific LacZ activity were seen in gall bladder and salivary gland while specific signals were obvious in lung (Fig 16). LacZ expression in kidney section was localized to renal tubules and strong signals in glomeruli and renal papilla in anterior of inner medulla (Fig 17). Secretive cells of glandular stomach and columnar absorptive region of intestinal villus were LacZ positive (Fig 18).

## Conclusions

Systematic LacZ staining of developing embryos and adult tissues has identified broad expression of *Dip2a* gene. Expression in embryo and adult is universally distributed among the neuron system. Very strong and broad expression is also found in male reproductive system. Less broad expression are seen in female reproductive, kidney, lung, liver and circulatory system. This information is very important in prediction and investigation of functional roles of *Dip2a* gene. The insertion of *LacZ* into the first exon disrupted *Dip2a* gene expression and function but no phenotypes were obvious.

## Discussion

### LacZ expression correlates with real time PCR results

**Fig 19:**
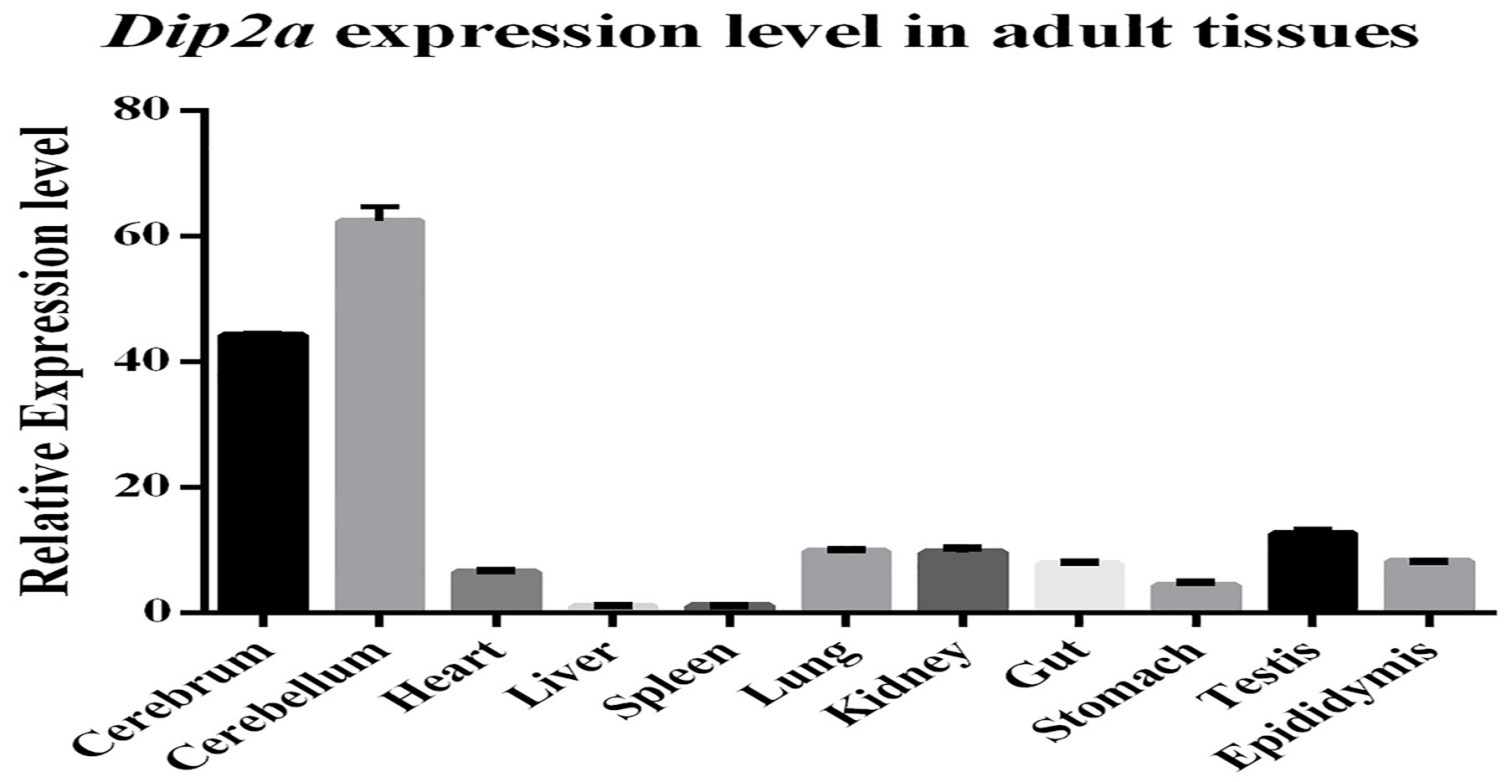
Real-time PCR from total RNA of 8 week old tissues. Relative mRNA levels were calculated using ΔΔ^Ct^ method. Data shown are representative of at least 3 independent repeats and expressed as the mean ± s.d.

*Dip2a-LacZ* allele was generated using the CRISPR/Cas9 system [4]. We performed systematic analysis of gene expression of *Dip2a* gene using *Dip2a-LacZ* mouse. We validated LacZ staining results with *Dip2a* gene expression results by quantitative real-time PCR (Fig 19). The results are highly correlated in corresponding tissues. The lacZ expression pattern can faithfully represent endogenous expression of *Dip2a* gene and can demonstrate more details in a cell type-specific fashion. Expression pattern of *Dip2a* ^*LacZ-neo/+*^ and *Dip2a* ^*LacZ/+*^ mice are indistinguishable in both embryos and adult tissues inspected, suggesting neomycin cassette has limited effect in this case [8].

### Broad neuronal expression may indicate potential neurological roles of *Dip2a* gene

Synapse formation and axon guidance are important events to establish functional neuron circuits [9]. Previous studies have suggested synapse formation and axon guidance are mediated by distinct molecular processes [9]. Studies have suggested that *Dip2a* may regulate synapse formation and axon path finding [2]. *Dip2a-LacZ* is highly expressed in neuronal cells such as dorsal root ganglion, retinal ganglion cells, Purkinje cell layer of cerebellum and granular cells of the dentate gyrus in hippocampus including cerebrum forebrain neurons (Fig 4). This finding supports previous reports of *Dip2a* signaling in axon guidance and potential role in neuronal function. Coexpression of *Dip2a* with other strongly expressed genes in central neuron systems may provide insight into how axon guidance and synapse formation are coordinated at molecular level. *Dip2a* was initially identified by yeast two-hybrid screening using a transcription factor protein *Drosophila Disco* [2]. Mutations at this locus can cause abnormal neuronal connections in visual system [10]. This phenotype is supported by the high LacZ expression in retina (Fig 7).

Broad expression of *Dip2a* in developing brain and central neuron systems in adult support its roles in multiple neurons (Figs 1–4). As stated previously from bioinformatics analysis, *Dip2a* is a type I receptor molecule with three binding domains, DMAP, CaiC and AMP, raising speculation that DIP2A might have a role in ligand-receptor interaction and specification of synapse formation. AMP-binding domains are found in protein families including long chain fatty acid CoA ligase, acetyl CoA synthase and other related synthases [1]. These enzymes play critical roles in metabolism and synthesis of neurotransmitters. Fatty acid metabolism through CoA synthase is important in brain development and function. Mutations in Fatty acid long-chain family member 4 (ACSL4) leads to nonsyndromic X-linked mental retardation [11].

### *Dip2a* expression in reproductive system

*Dip2a* gene is strongly expressed in male reproductive system. Based on the intensity of signal, we speculate *Dip2a* may have stronger impact on spermatogenesis and sperm function. *Dip2a* may participate in ovule development and function as well based on its expression. Specific expression in endometrium cells may indicate the role of *Dip2a* in implantation process of uterus.

### *Dip2a* in vascular system

Our data show that *Dip2a-LacZ* is expressed in vascular system including veins, arteries and cardiomyocyte. Upon sectioning, we observed LacZ expression in endothelial cells and smooth muscle cells. Strong signals of *Dip2a* were observed in varieties of veins, including saphenous vein of limb vasculature, tongue vein, tail vein and tail skin vein (Fig 9). This observation and earlier reports [12] suggests potential role of *Dip2a* gene in vasculature. Damaged endothelium can cause endothelial cell detachment and result in increased circulating endothelial cells, a causative factor of male hypogonadism. DIP2A is expressed on endothelial cell surface and has been suggested to be receptor of FSTL1 [13]. Knockdown of *Dip2a* expression using small interfering RNA reduced FSTL1 binding. This could be the causative to malfunctioning of endothelial cell. FSTL1 has protective effect on ischemia-reperfusion injury in muscle and heart tissue [14–16]. Protective effect against apoptosis in cardiac ischemia was mediated by activation of DIP2A. Strong and universal LacZ expression in cardiomyocytes may suggest potential roles of *Dip2a* in heart function.

### Potential role of *Dip2a* in other tissues

In addition to nervous, reproductive and cardiovascular system, we have identified strong expression of *Dip2a* in many other organs, including digestive, respiratory and urinary system. Cytological investigations are ongoing to identify cell type-specific distribution of *Dip2a* gene expression and to elucidate possible functions of *Dip2a* gene in these organs. It has been reported that both mouse and human express *Dip2a* in kidney and DIP2A is potential receptor of FSTL1 [13]. DIP2A is required for anti-apoptotic and promigratory effects of FSTL1 and activation of Akt in endothelial cells. Adam has reported coexpression patterns of *Fstl1* and *Dip2a* in adult mouse kidney [17]. Relatively weak expression of FSTLI and DIP2A was observed in human kidney [17]. Strong kidney expression in glomeruli and renal papilla were detected using *Dip2a-lacZ* mice.

### Phenotype analysis

The insertion of LacZ fragment into first exon of *Dip2a* gene was designed to impair *Dip2a* gene translation and its biological function, but viability of *Dip2a* ^*LacZ/+*^ and *Dip2a* ^*LacZ/LacZ*^ mice was not affected (S1 Table). Postnatal viability was also confirmed in backcrossing of *Dip2a-LacZ* mice with C57BL/6J, a switch of genetic background towards C57BL/6J. Similar result was confirmed in complete knockout of 65kb *Dip2a* genomic sequence using CRISPR/Cas9 system [4]. No obvious phenotype was instantly identifiable although we cannot exclude subtle physiological changes without careful measurement (S1 Fig). Some phenotypes may need conditional challenges. We have found high correlations of *Dip2a* and *Fstl1* gene expression in neuronal, reproductive and vascular systems and as well as in heart and kidney. Our results do not contradict the report that FSTL1 associates with DIP2A protein and signal through DIP2A. DIP2B has been reported to be associated with human neurocognitive disorder [18]. We suspect compensation roles of *Dip2b* and *Dip2c* from *Dip2* gene family may mask phenotype analysis. Double and triple knockout of *Dip2* genes are being undertaken to uncover the biological functions of DIP2 family.

## Supporting Information

S1 Fig *Dip2a-LacZ* staining of hets and homo brain. Adult brain sections from *Dip2a* ^*LacZ/WT*^ and *Dip2a* ^*LacZ/LacZ*^ mice were compared side by side. No obvious differences were observed except stronger staining in homo.
(TIF)

S1 Table Postnatal viability of *Dip2a* ^*LacZ/LacZ*^ mice. Genotyping result obtained at 3 weeks postnatal reveals normal Mendelian ratio from *Dip2a* ^*LacZ/WT*^ inbred mating.
(PDF)
