# Supplementary figures and images for: Expression Patterns and Potential Biological Roles of Dip2a
Source: PLoS One. 2015 Nov 25;10(11):e0143284. doi: 10.1371/journal.pone.0143284 (PMC4659570; doi:10.1371/journal.pone.0143284)

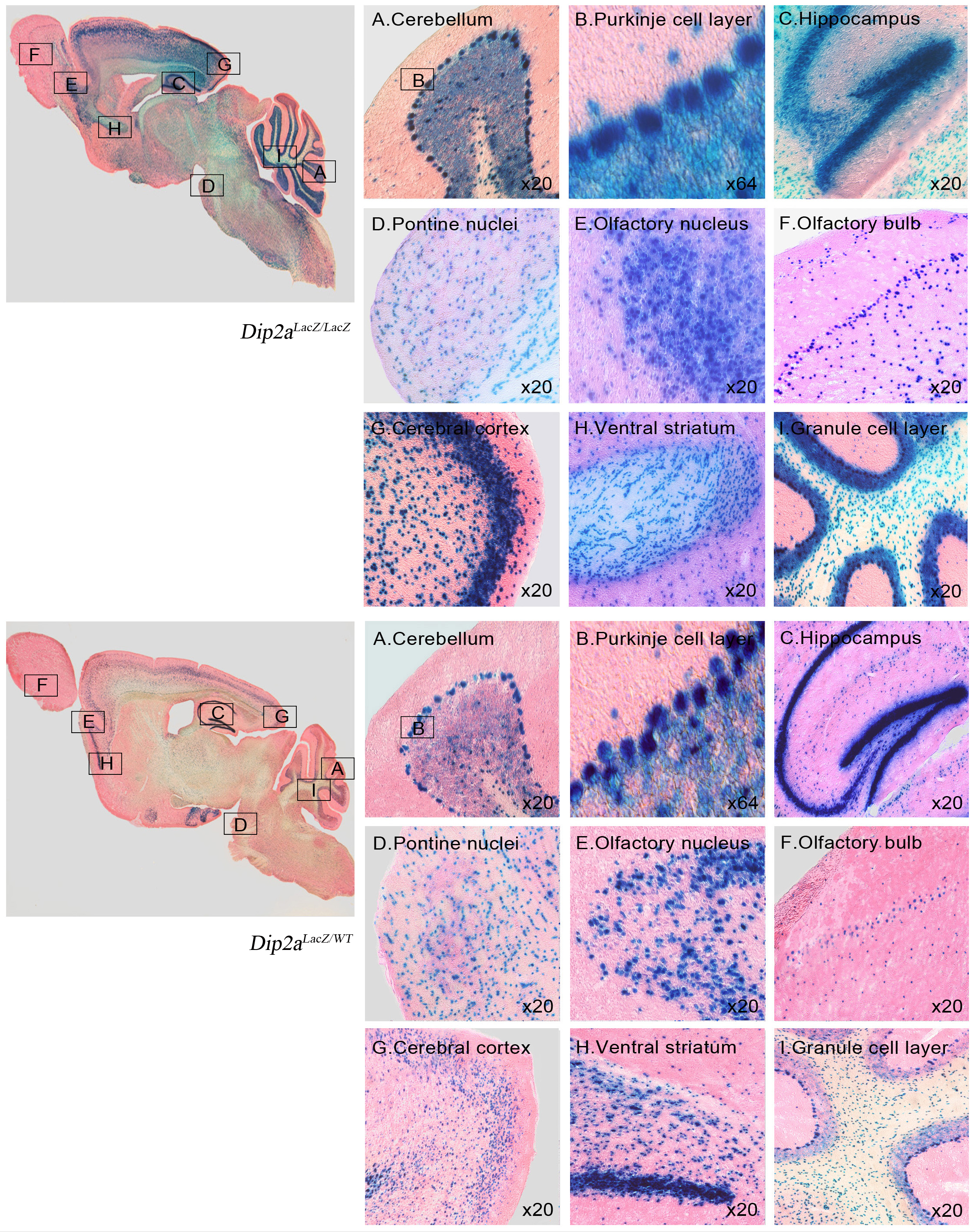

Supplement: S1 Fig — Adult brain sections from Dip2a LacZ/WT and Dip2a LacZ/LacZ mice were compared side by side. No obvious differences were observed except stronger staining in homo. (TIF) [file pone.0143284.s001.tif]
