# Supplementary material for: Expression Patterns and Potential Biological Roles of Dip2a
Source: PLoS One. 2015 Nov 25;10(11):e0143284. doi: 10.1371/journal.pone.0143284 (PMC4659570; doi:10.1371/journal.pone.0143284)
Supplement: S1 Table — Genotyping result obtained at 3 weeks postnatal reveals normal Mendelian ratio from Dip2a LacZ/WT inbred mating. (PDF) [file pone.0143284.s002.pdf]

MATING	*Dip2a*$^{WT/WT}$	*Dip2a*$^{LacZ/WT}$	*Dip2a*$^{LacZ/LacZ}$
**HET × HET**	2	3	4
**HET × HET**	4	6	6
**HET × HET**	7	11	3
**HET × HET**	8	11	3
**HET × HET**	1	4	3
**HET × HET**	12	24	11
**HET × HET**	8	9	5
**HET × HET**	5	28	14
TOTAL	**47**	**96**	**49**
